# ID 154 - Blog Rede NatJus: informações estratégicas para apoiar o Judiciário na tomada de decisão em saúde

**DOI:** 10.5327/2237-9622.2025.v34s1.154

**Published:** 2025-11-25

**Authors:** Verônica Colpani, Verônica Colpani, Isabela Porto de Toledo, Ana Luiza Cabrera Martimbianco, Carolina de Oliveira Cruz Latorraca, Camila Monteiro Cruz, Cecília Menezes Farinasso, Patrícia do Carmo Silva Parreira, Rafael Leite Pacheco, Roberta Borges Silva, Rachel Riera

**Keywords:** disseminação de informação, comunicação e divulgação científica, medicina baseada em evidências, avaliação da tecnologia em saúde, blog

## Abstract

**Introdução:**

Entre 2008 e 2017, o número de ações judiciais relacionadas à saúde no Brasil aumentou 130% acompanhado por aumento de 50% nas ações judiciais por todas as causas. Diversas estratégias têm sido implementadas para entender o cenário e identificar medidas para melhorar o processo de judicialização da saúde no Brasil. O objetivo deste estudo consiste em apresentar a implementação e os dados de acessos de um blog focado em conteúdos de medicina baseada em evidências (MBE) e avaliação de tecnologias em saúde (ATS) para profissionais de saúde e operadores do direito envolvidos com a judicialização da saúde no Brasil.

**Método:**

Estudo descritivo e analítico sobre a iniciativa de disseminação "Blog Rede NATJus" (https://redenatjus.org.br/blog/), desenvolvido pelo Nats do Hospital Sírio-Libanês (Nats-HSL), em parceria com o Departamento de Gestão das Demandas em Judicialização na Saúde (Djud/SE/MS) e o Conselho Nacional de Justiça, por meio do Programa de Apoio ao Desenvolvimento Institucional do Sistema Único de Saúde (Proadi-SUS).

**Resultados:**

O blog Rede NatJus foi lançado oficialmente em 3 de outubro de 2019 e, além de MBE e ATS, aborda questões relacionadas à incorporação de tecnologias pelo SUS, à judicialização da saúde e cursos/eventos relacionados. As postagens são disponibilizadas semanalmente e as mais relevantes também têm sido enviadas como newsletters para uma lista de e-mails de, aproximadamente, 13 mil usuários (previamente identificados ou registrados espontaneamente na homepage do blog). No período de 1º de janeiro de 2020 até 6 de setembro de 2024, foram publicados 378 posts e, segundo os dados do Google Analytics, 92,6% dos acessos foram do Brasil, 5,3% dos EUA e 0,5% da França. Os principais canais de acesso foram 35,2% via busca orgânica, 32,7% direto no site e 24,5% por e-mail (newsletter). O blog possui cinco categorias de postagens, que norteiam os temas dos posts, sendo elas: Agenda, ATS-Evidências, Judicialização em Saúde, Núcleos de Apoio Técnico ao Judiciário (NatJus), e Sistema e-NatJus. Entre essas categorias, as postagens mais acessadas foram aquelas sobre os NatJus, seguidas por ATS-Evidências e os posts sobre agenda. Entre 3 de outubro de 2019 a 6 de setembro de 2024, os três posts mais acessados abordaram: a ferramenta on-line de consulta de preço de medicamentos da SCMED; o III Fórum de Direito e Saúde; e o curso de avaliação crítica de estudos primários e revisões sistemáticas promovido pelo Nats-HSL.

**Conclusão:**

A divulgação de conteúdos sobre MBE/ATS por meio do blog estabeleceu um canal inovador de comunicação com os NatJus, além de atingir operadores do direito e profissionais de saúde interessados no assunto. A Rede NatJus destaca-se como uma fonte confiável e de fácil compreensão, fornecendo informações que podem aprimorar a eficácia das decisões judiciais no País, ao promover o uso de tecnologias comprovadamente eficazes e seguras, e descartando aquelas que são ineficazes, prejudiciais ou com eficácia e segurança incertas.

